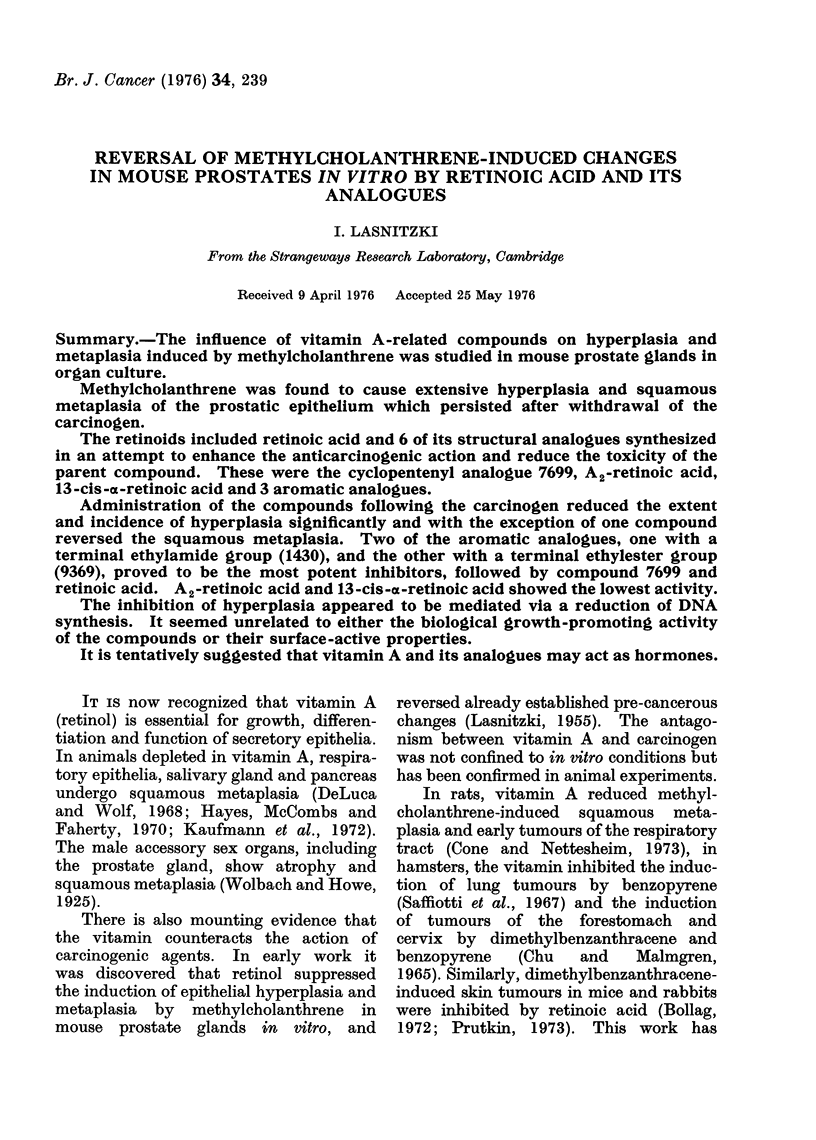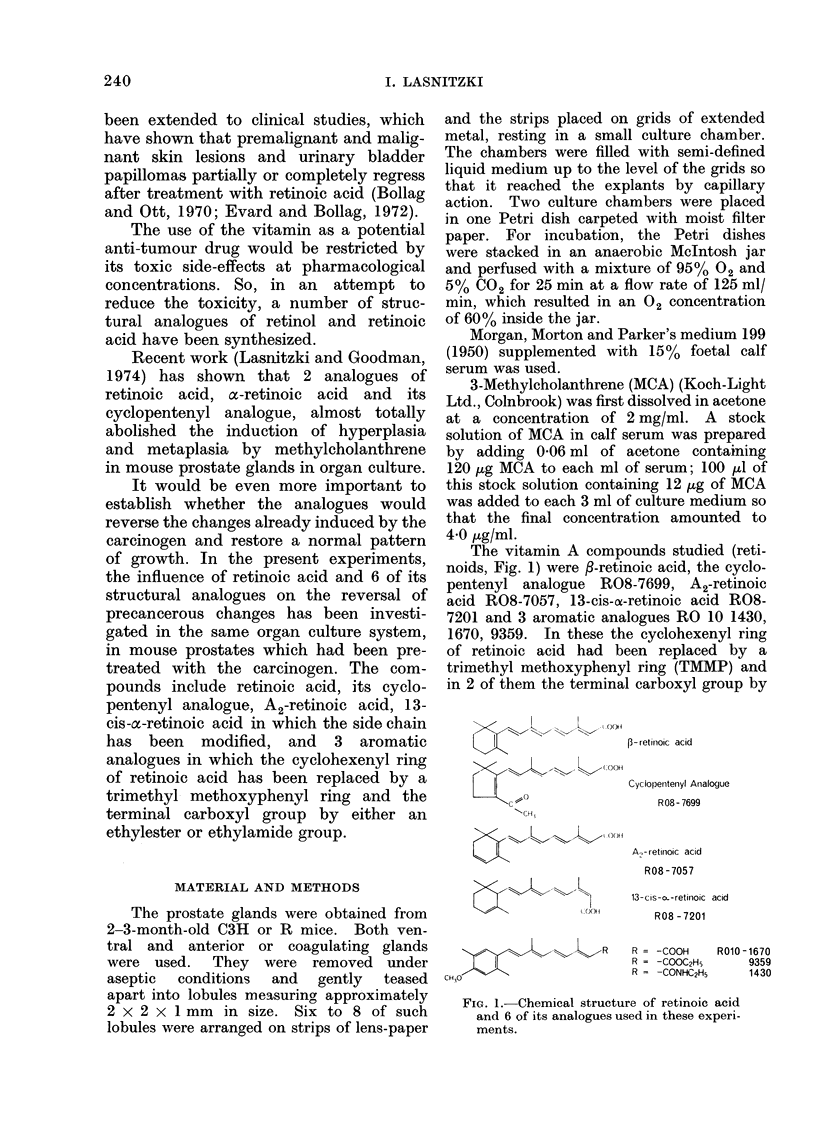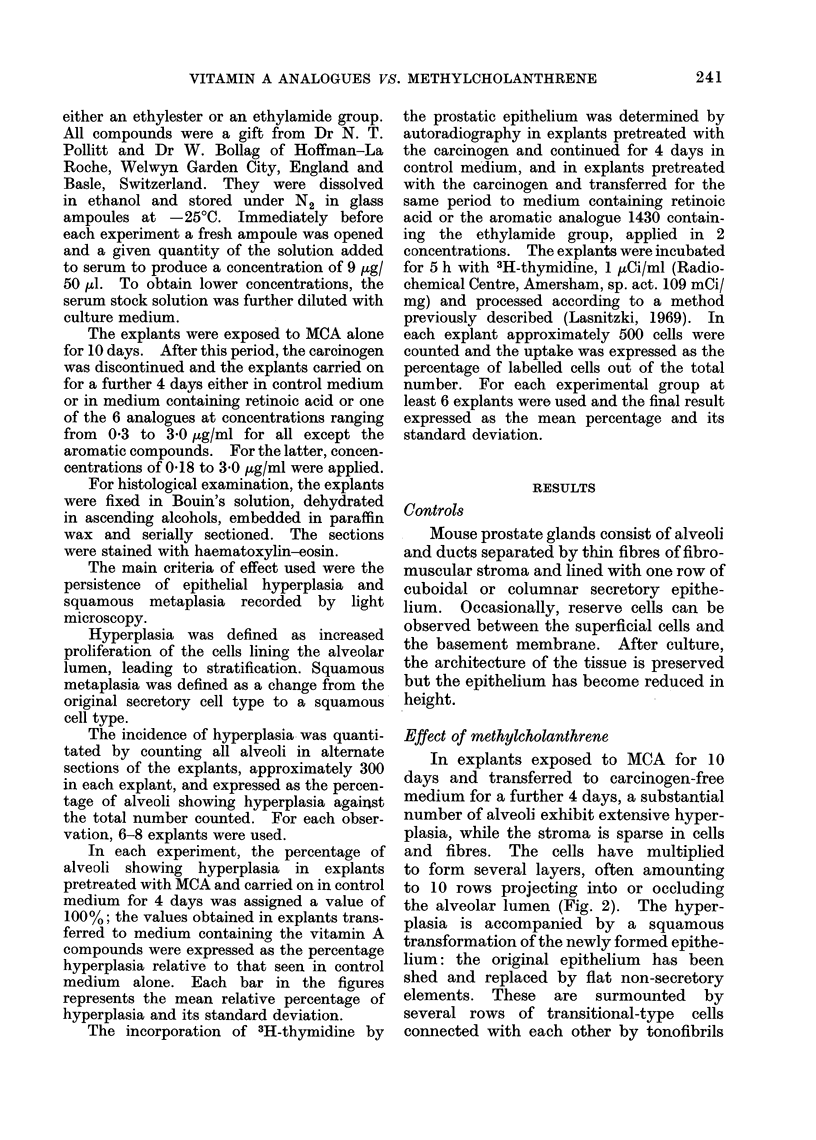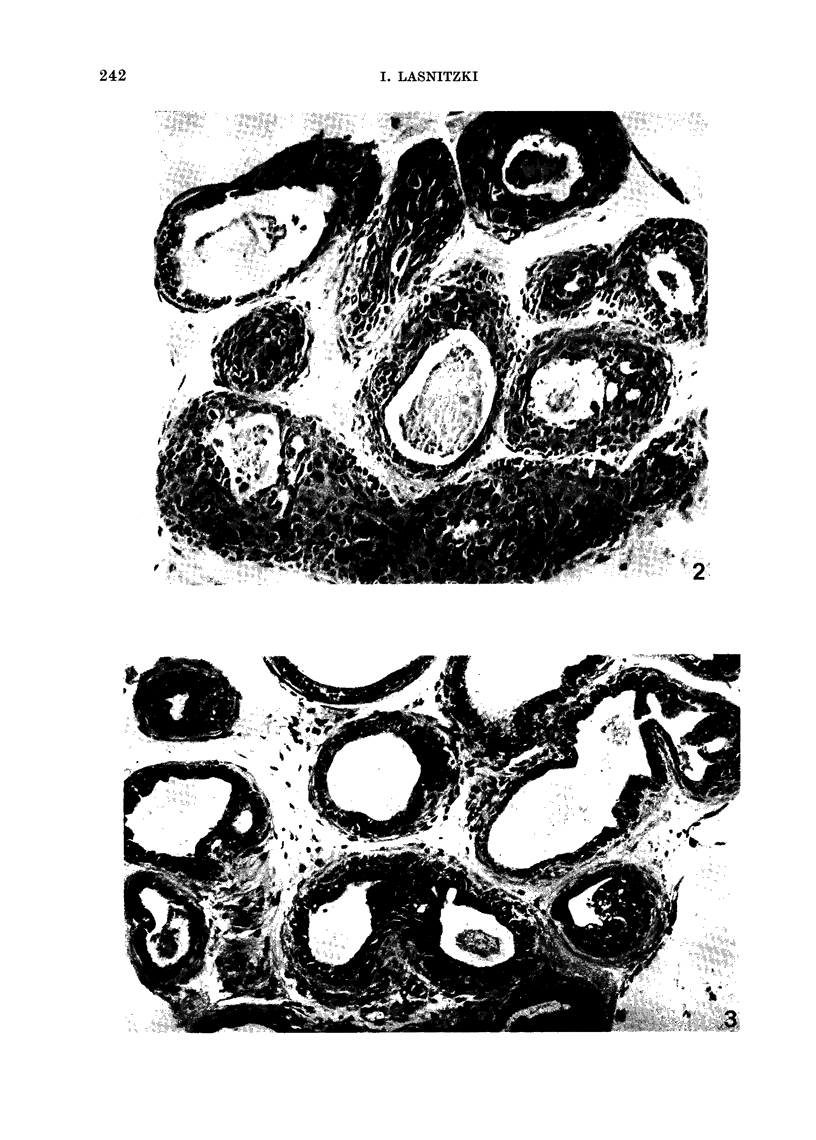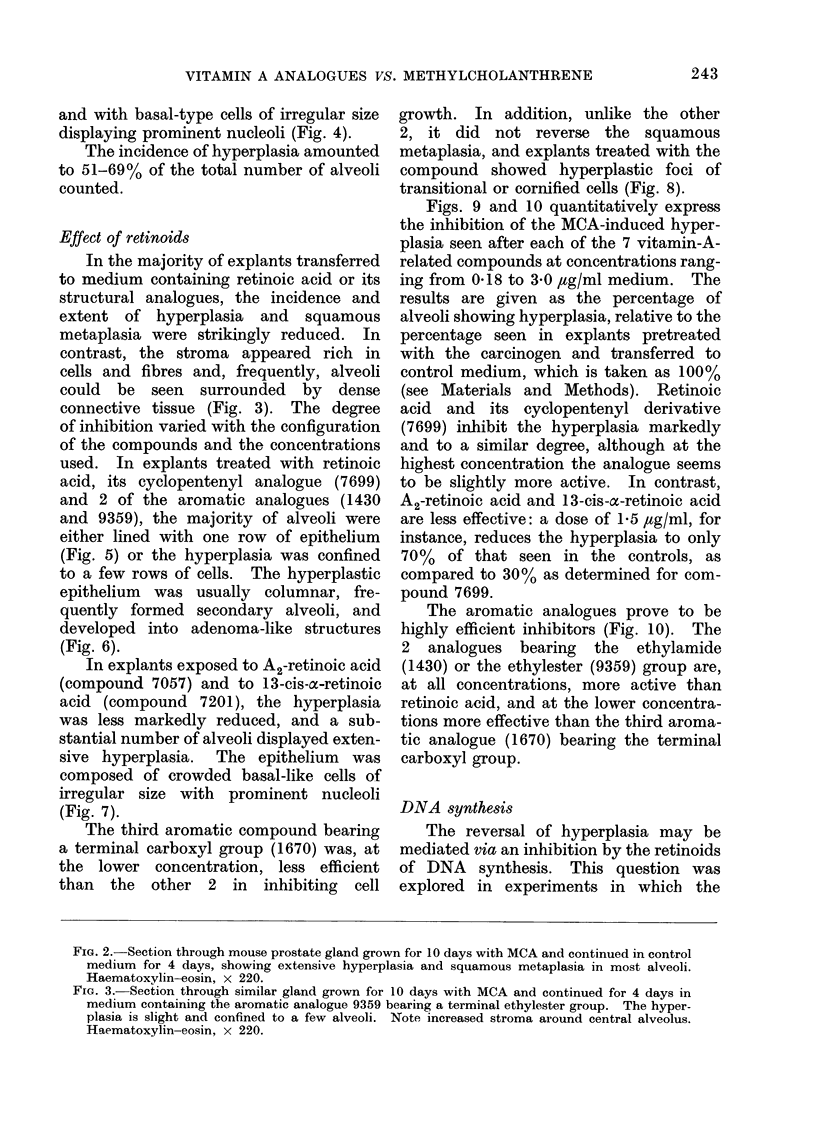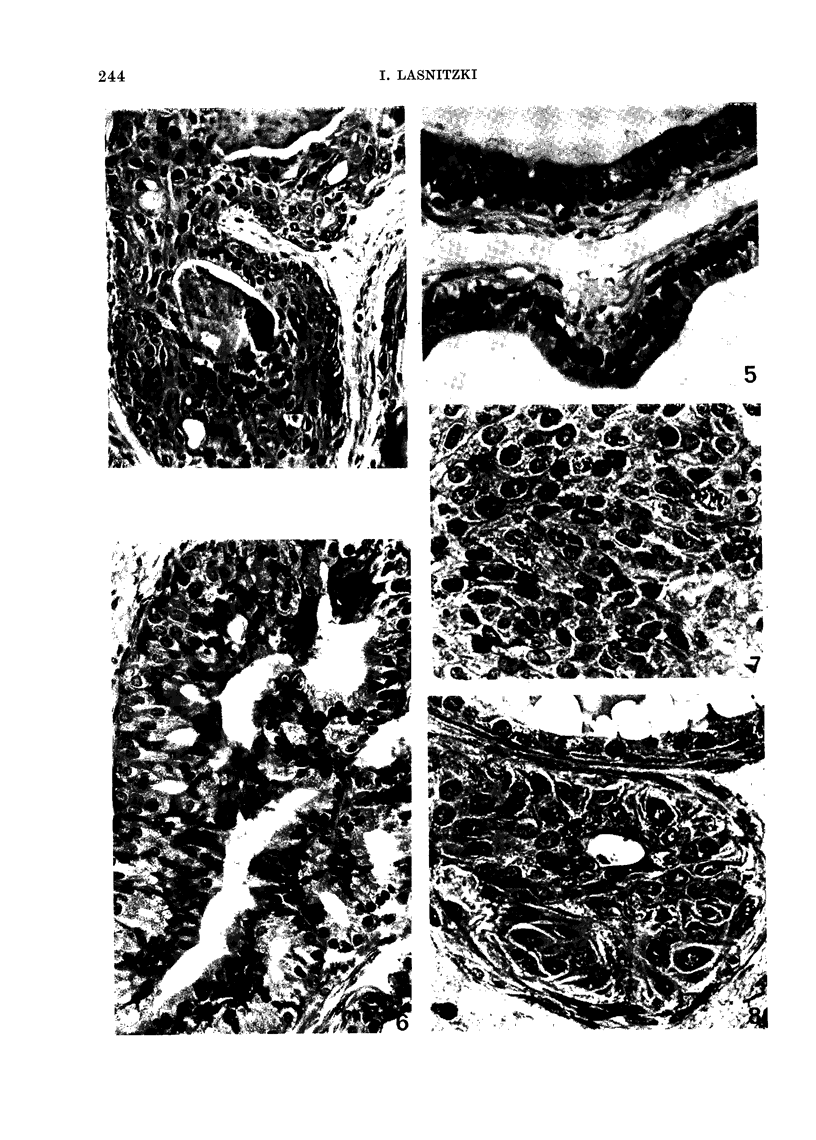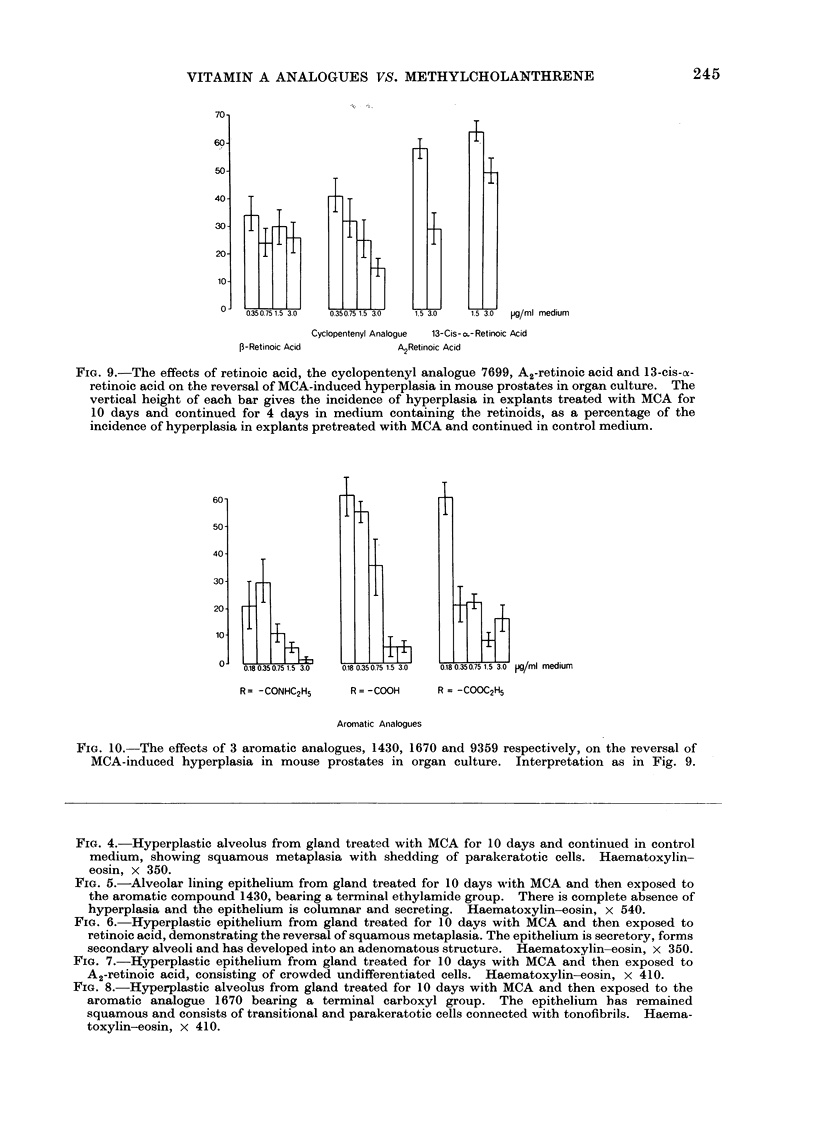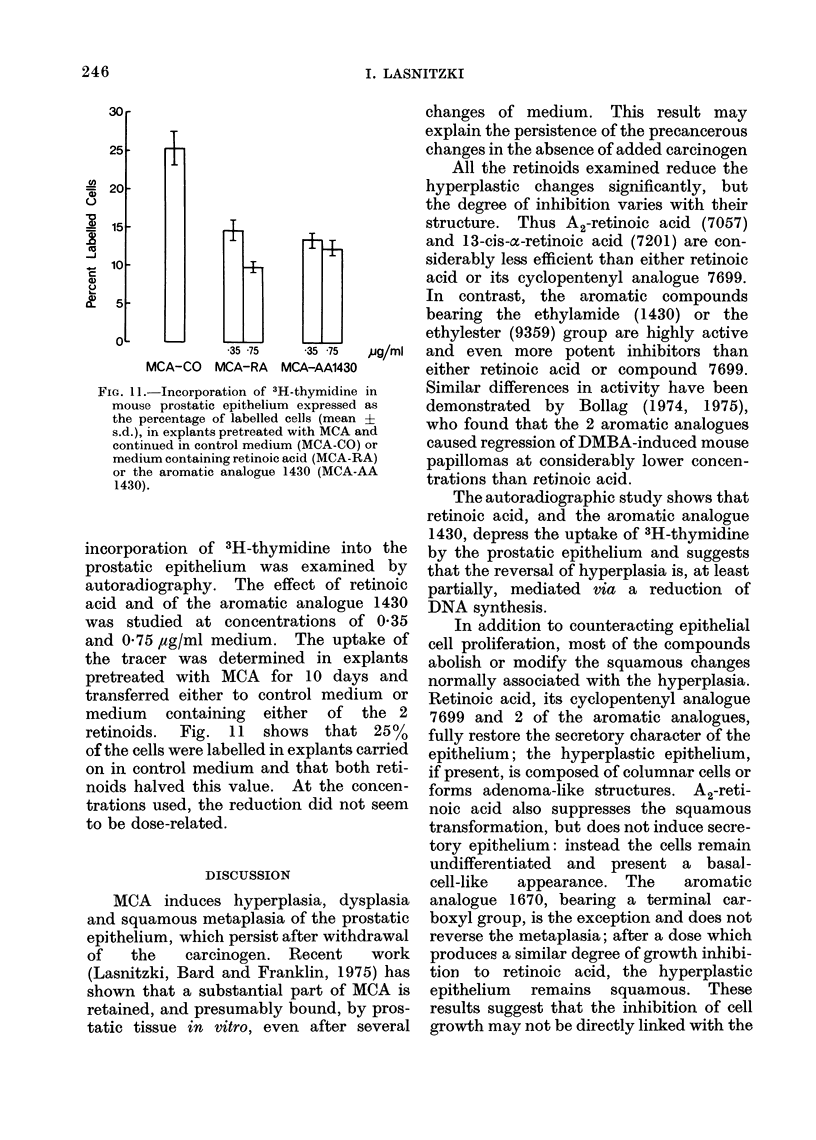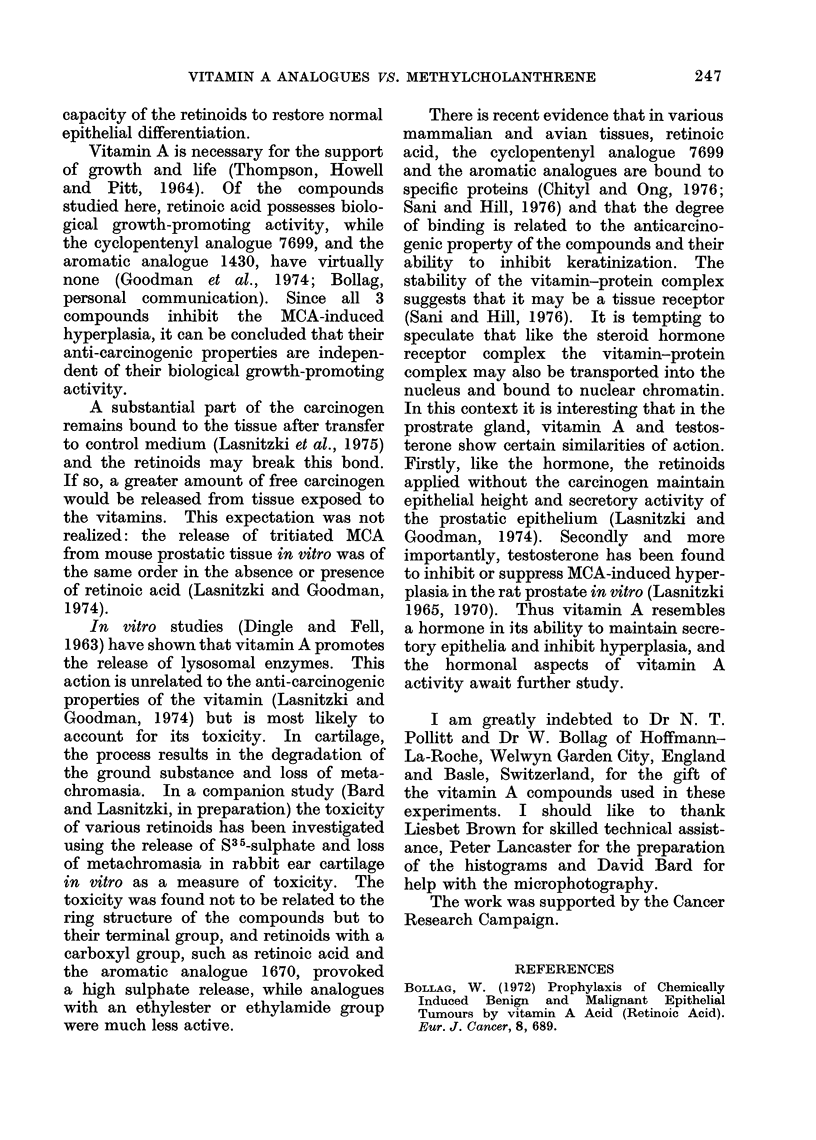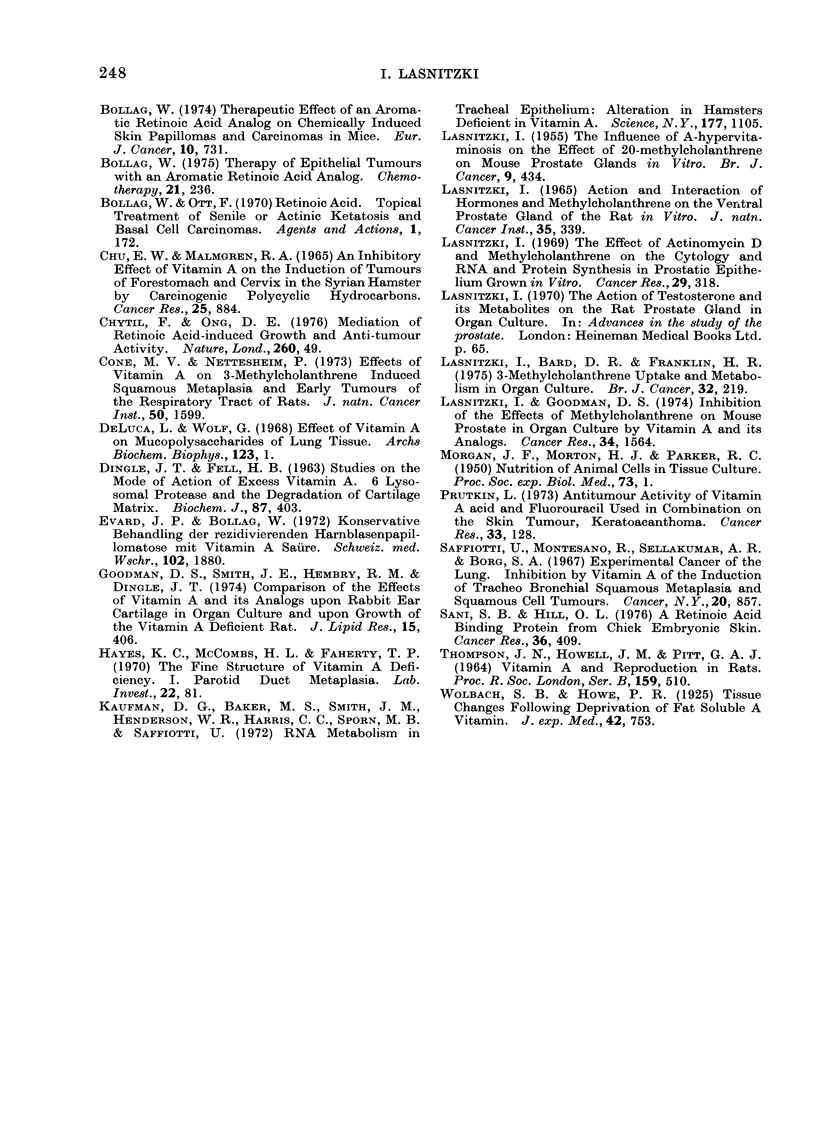# Reversal of methylcholanthrene-induced changes in mouse prostates in vitro by retinoic acid and its analogues.

**DOI:** 10.1038/bjc.1976.158

**Published:** 1976-09

**Authors:** I. Lasnitzki

## Abstract

**Images:**


*Br. J. Cancer* (1976) **34**, 239

### REVERSAL OF METHYLCHOLANTHRENE-INDUCED CHANGES IN MOUSE PROSTATES *IN VITRO* BY RETINOIC ACID AND ITS ANALOGUES

I. LASNITZKI

*From the Strangeways Research Laboratory, Cambridge*

Received 9 April 1976 Accepted 25 May 1976

**Summary.—The influence of vitamin A-related compounds on hyperplasia and metaplasia induced by methylcholanthrene was studied in mouse prostate glands in organ culture.**

**Methylcholanthrene was found to cause extensive hyperplasia and squamous metaplasia of the prostatic epithelium which persisted after withdrawal of the carcinogen.**

**The retinoids included retinoic acid and 6 of its structural analogues synthesized in an attempt to enhance the anticarcinogenic action and reduce the toxicity of the parent compound. These were the cyclopentenyl analogue 7699, $A_2$-retinoic acid, 13-cis-α-retinoic acid and 3 aromatic analogues.**

**Administration of the compounds following the carcinogen reduced the extent and incidence of hyperplasia significantly and with the exception of one compound reversed the squamous metaplasia. Two of the aromatic analogues, one with a terminal ethylamide group (1430), and the other with a terminal ethylester group (9369), proved to be the most potent inhibitors, followed by compound 7699 and retinoic acid. $A_2$-retinoic acid and 13-cis-α-retinoic acid showed the lowest activity.**

**The inhibition of hyperplasia appeared to be mediated via a reduction of DNA synthesis. It seemed unrelated to either the biological growth-promoting activity of the compounds or their surface-active properties.**

**It is tentatively suggested that vitamin A and its analogues may act as hormones.**

IT IS now recognized that vitamin A (retinol) is essential for growth, differentiation and function of secretory epithelia. In animals depleted in vitamin A, respiratory epithelia, salivary gland and pancreas undergo squamous metaplasia (DeLuca and Wolf, 1968; Hayes, McCombs and Faherty, 1970; Kaufmann *et al.*, 1972). The male accessory sex organs, including the prostate gland, show atrophy and squamous metaplasia (Wolbach and Howe, 1925).

There is also mounting evidence that the vitamin counteracts the action of carcinogenic agents. In early work it was discovered that retinol suppressed the induction of epithelial hyperplasia and metaplasia by methylcholanthrene in mouse prostate glands *in vitro*, and reversed already established pre-cancerous changes (Lasnitzki, 1955). The antagonism between vitamin A and carcinogen was not confined to *in vitro* conditions but has been confirmed in animal experiments.

In rats, vitamin A reduced methylcholanthrene-induced squamous metaplasia and early tumours of the respiratory tract (Cone and Nettesheim, 1973), in hamsters, the vitamin inhibited the induction of lung tumours by benzopyrene (Saffiotti *et al.*, 1967) and the induction of tumours of the forestomach and cervix by dimethylbenzanthracene and benzopyrene (Chu and Malmgren, 1965). Similarly, dimethylbenzanthracene-induced skin tumours in mice and rabbits were inhibited by retinoic acid (Bollag, 1972; Prutkin, 1973). This work has been extended to clinical studies, which have shown that premalignant and malignant skin lesions and urinary bladder papillomas partially or completely regress after treatment with retinoic acid (Bollag and Ott, 1970; Evard and Bollag, 1972).

The use of the vitamin as a potential anti-tumour drug would be restricted by its toxic side-effects at pharmacological concentrations. So, in an attempt to reduce the toxicity, a number of structural analogues of retinol and retinoic acid have been synthesized.

Recent work (Lasnitzki and Goodman, 1974) has shown that 2 analogues of retinoic acid, $\alpha$-retinoic acid and its cyclopentenyl analogue, almost totally abolished the induction of hyperplasia and metaplasia by methylcholanthrene in mouse prostate glands in organ culture.

It would be even more important to establish whether the analogues would reverse the changes already induced by the carcinogen and restore a normal pattern of growth. In the present experiments, the influence of retinoic acid and 6 of its structural analogues on the reversal of precancerous changes has been investigated in the same organ culture system, in mouse prostates which had been pretreated with the carcinogen. The compounds include retinoic acid, its cyclopentenyl analogue, $A_2$-retinoic acid, 13-cis-$\alpha$-retinoic acid in which the side chain has been modified, and 3 aromatic analogues in which the cyclohexenyl ring of retinoic acid has been replaced by a trimethyl methoxyphenyl ring and the terminal carboxyl group by either an ethylester or ethylamide group.

### MATERIAL AND METHODS

The prostate glands were obtained from 2–3-month-old C3H or R mice. Both ventral and anterior or coagulating glands were used. They were removed under aseptic conditions and gently teased apart into lobules measuring approximately 2 × 2 × 1 mm in size. Six to 8 of such lobules were arranged on strips of lens-paper and the strips placed on grids of extended metal, resting in a small culture chamber. The chambers were filled with semi-defined liquid medium up to the level of the grids so that it reached the explants by capillary action. Two culture chambers were placed in one Petri dish carpeted with moist filter paper. For incubation, the Petri dishes were stacked in an anaerobic McIntosh jar and perfused with a mixture of 95% $O_2$ and 5% $CO_2$ for 25 min at a flow rate of 125 ml/min, which resulted in an $O_2$ concentration of 60% inside the jar.

Morgan, Morton and Parker's medium 199 (1950) supplemented with 15% foetal calf serum was used.

3-Methylcholanthrene (MCA) (Koch-Light Ltd., Colnbrook) was first dissolved in acetone at a concentration of 2 mg/ml. A stock solution of MCA in calf serum was prepared by adding 0·06 ml of acetone containing 120 $\mu$g MCA to each ml of serum; 100 $\mu$l of this stock solution containing 12 $\mu$g of MCA was added to each 3 ml of culture medium so that the final concentration amounted to 4·0 $\mu$g/ml.

The vitamin A compounds studied (retinoids, Fig. 1) were $\beta$-retinoic acid, the cyclopentenyl analogue RO8-7699, $A_2$-retinoic acid RO8-7057, 13-cis-$\alpha$-retinoic acid RO8-7201 and 3 aromatic analogues RO 10 1430, 1670, 9359. In these the cyclohexenyl ring of retinoic acid had been replaced by a trimethyl methoxyphenyl ring (TMMP) and in 2 of them the terminal carboxyl group by

β-retinoic acid

Cyclopentenyl Analogue
R08-7699

A2-retinoic acid
R08-7057

13-cis-α-retinoic acid

R08-7201

R = -COOH R010-1670
R = -COOC2H5 9359
R = -CONHC2H5 1430

FIG. 1.—Chemical structure of retinoic acid and 6 of its analogues used in these experiments.

either an ethylester or an ethylamide group. All compounds were a gift from Dr N. T. Pollitt and Dr W. Bollag of Hoffman–La Roche, Welwyn Garden City, England and Basle, Switzerland. They were dissolved in ethanol and stored under $N_2$ in glass ampoules at −25°C. Immediately before each experiment a fresh ampoule was opened and a given quantity of the solution added to serum to produce a concentration of 9 $\mu$g/50 $\mu$l. To obtain lower concentrations, the serum stock solution was further diluted with culture medium.

The explants were exposed to MCA alone for 10 days. After this period, the carcinogen was discontinued and the explants carried on for a further 4 days either in control medium or in medium containing retinoic acid or one of the 6 analogues at concentrations ranging from 0·3 to 3·0 $\mu$g/ml for all except the aromatic compounds. For the latter, concentrations of 0·18 to 3·0 $\mu$g/ml were applied.

For histological examination, the explants were fixed in Bouin's solution, dehydrated in ascending alcohols, embedded in paraffin wax and serially sectioned. The sections were stained with haematoxylin–eosin.

The main criteria of effect used were the persistence of epithelial hyperplasia and squamous metaplasia recorded by light microscopy.

Hyperplasia was defined as increased proliferation of the cells lining the alveolar lumen, leading to stratification. Squamous metaplasia was defined as a change from the original secretory cell type to a squamous cell type.

The incidence of hyperplasia was quantitated by counting all alveoli in alternate sections of the explants, approximately 300 in each explant, and expressed as the percentage of alveoli showing hyperplasia against the total number counted. For each observation, 6–8 explants were used.

In each experiment, the percentage of alveoli showing hyperplasia in explants pretreated with MCA and carried on in control medium for 4 days was assigned a value of 100%; the values obtained in explants transferred to medium containing the vitamin A compounds were expressed as the percentage hyperplasia relative to that seen in control medium alone. Each bar in the figures represents the mean relative percentage of hyperplasia and its standard deviation.

The incorporation of $^{3}$H-thymidine by the prostatic epithelium was determined by autoradiography in explants pretreated with the carcinogen and continued for 4 days in control medium, and in explants pretreated with the carcinogen and transferred for the same period to medium containing retinoic acid or the aromatic analogue 1430 containing the ethylamide group, applied in 2 concentrations. The explants were incubated for 5 h with $^{3}$H-thymidine, 1 $\mu$Ci/ml (Radiochemical Centre, Amersham, sp. act. 109 mCi/mg) and processed according to a method previously described (Lasnitzki, 1969). In each explant approximately 500 cells were counted and the uptake was expressed as the percentage of labelled cells out of the total number. For each experimental group at least 6 explants were used and the final result expressed as the mean percentage and its standard deviation.

### RESULTS

### *Controls*

Mouse prostate glands consist of alveoli and ducts separated by thin fibres of fibromuscular stroma and lined with one row of cuboidal or columnar secretory epithelium. Occasionally, reserve cells can be observed between the superficial cells and the basement membrane. After culture, the architecture of the tissue is preserved but the epithelium has become reduced in height.

### *Effect of methylcholanthrene*

In explants exposed to MCA for 10 days and transferred to carcinogen-free medium for a further 4 days, a substantial number of alveoli exhibit extensive hyperplasia, while the stroma is sparse in cells and fibres. The cells have multiplied to form several layers, often amounting to 10 rows projecting into or occluding the alveolar lumen (Fig. 2). The hyperplasia is accompanied by a squamous transformation of the newly formed epithelium: the original epithelium has been shed and replaced by flat non-secretory elements. These are surmounted by several rows of transitional-type cells connected with each other by tonofibrils

2

3

and with basal-type cells of irregular size displaying prominent nucleoli (Fig. 4).

The incidence of hyperplasia amounted to 51–69% of the total number of alveoli counted.

*Effect of retinoids*

In the majority of explants transferred to medium containing retinoic acid or its structural analogues, the incidence and extent of hyperplasia and squamous metaplasia were strikingly reduced. In contrast, the stroma appeared rich in cells and fibres and, frequently, alveoli could be seen surrounded by dense connective tissue (Fig. 3). The degree of inhibition varied with the configuration of the compounds and the concentrations used. In explants treated with retinoic acid, its cyclopentenyl analogue (7699) and 2 of the aromatic analogues (1430 and 9359), the majority of alveoli were either lined with one row of epithelium (Fig. 5) or the hyperplasia was confined to a few rows of cells. The hyperplastic epithelium was usually columnar, frequently formed secondary alveoli, and developed into adenoma-like structures (Fig. 6).

In explants exposed to $A_2$-retinoic acid (compound 7057) and to 13-cis-$\alpha$-retinoic acid (compound 7201), the hyperplasia was less markedly reduced, and a substantial number of alveoli displayed extensive hyperplasia. The epithelium was composed of crowded basal-like cells of irregular size with prominent nucleoli (Fig. 7).

The third aromatic compound bearing a terminal carboxyl group (1670) was, at the lower concentration, less efficient than the other 2 in inhibiting cell growth. In addition, unlike the other 2, it did not reverse the squamous metaplasia, and explants treated with the compound showed hyperplastic foci of transitional or cornified cells (Fig. 8).

Figs. 9 and 10 quantitatively express the inhibition of the MCA-induced hyperplasia seen after each of the 7 vitamin-A-related compounds at concentrations ranging from 0·18 to 3·0 $\mu$g/ml medium. The results are given as the percentage of alveoli showing hyperplasia, relative to the percentage seen in explants pretreated with the carcinogen and transferred to control medium, which is taken as 100% (see Materials and Methods). Retinoic acid and its cyclopentenyl derivative (7699) inhibit the hyperplasia markedly and to a similar degree, although at the highest concentration the analogue seems to be slightly more active. In contrast, $A_2$-retinoic acid and 13-cis-$\alpha$-retinoic acid are less effective: a dose of 1·5 $\mu$g/ml, for instance, reduces the hyperplasia to only 70% of that seen in the controls, as compared to 30% as determined for compound 7699.

The aromatic analogues prove to be highly efficient inhibitors (Fig. 10). The 2 analogues bearing the ethylamide (1430) or the ethylester (9359) group are, at all concentrations, more active than retinoic acid, and at the lower concentrations more effective than the third aromatic analogue (1670) bearing the terminal carboxyl group.

*DNA synthesis*

The reversal of hyperplasia may be mediated *via* an inhibition by the retinoids of DNA synthesis. This question was explored in experiments in which the

---

FIG. 2.—Section through mouse prostate gland grown for 10 days with MCA and continued in control medium for 4 days, showing extensive hyperplasia and squamous metaplasia in most alveoli. Haematoxylin–eosin, × 220.

FIG. 3.—Section through similar gland grown for 10 days with MCA and continued for 4 days in medium containing the aromatic analogue 9359 bearing a terminal ethylester group. The hyperplasia is slight and confined to a few alveoli. Note increased stroma around central alveolus. Haematoxylin–eosin, × 220.

4

5

6

7

8

FIG. 9.—The effects of retinoic acid, the cyclopentenyl analogue 7699, $A_2$-retinoic acid and 13-cis-α-retinoic acid on the reversal of MCA-induced hyperplasia in mouse prostates in organ culture. The vertical height of each bar gives the incidence of hyperplasia in explants treated with MCA for 10 days and continued for 4 days in medium containing the retinoids, as a percentage of the incidence of hyperplasia in explants pretreated with MCA and continued in control medium.

FIG. 10.—The effects of 3 aromatic analogues, 1430, 1670 and 9359 respectively, on the reversal of MCA-induced hyperplasia in mouse prostates in organ culture. Interpretation as in Fig. 9.

---

FIG. 4.—Hyperplastic alveolus from gland treated with MCA for 10 days and continued in control medium, showing squamous metaplasia with shedding of parakeratotic cells. Haematoxylin–eosin, × 350.

FIG. 5.—Alveolar lining epithelium from gland treated for 10 days with MCA and then exposed to the aromatic compound 1430, bearing a terminal ethylamide group. There is complete absence of hyperplasia and the epithelium is columnar and secreting. Haematoxylin–eosin, × 540.

FIG. 6.—Hyperplastic epithelium from gland treated for 10 days with MCA and then exposed to retinoic acid, demonstrating the reversal of squamous metaplasia. The epithelium is secretory, forms secondary alveoli and has developed into an adenomatous structure. Haematoxylin–eosin, × 350.

FIG. 7.—Hyperplastic epithelium from gland treated for 10 days with MCA and then exposed to $A_2$-retinoic acid, consisting of crowded undifferentiated cells. Haematoxylin–eosin, × 410.

FIG. 8.—Hyperplastic alveolus from gland treated for 10 days with MCA and then exposed to the aromatic analogue 1670 bearing a terminal carboxyl group. The epithelium has remained squamous and consists of transitional and parakeratotic cells connected with tonofibrils. Haematoxylin–eosin, × 410.

FIG. 11.—Incorporation of $^3$H-thymidine in mouse prostatic epithelium expressed as the percentage of labelled cells (mean ± s.d.), in explants pretreated with MCA and continued in control medium (MCA-CO) or medium containing retinoic acid (MCA-RA) or the aromatic analogue 1430 (MCA-AA 1430).

incorporation of $^3$H-thymidine into the prostatic epithelium was examined by autoradiography. The effect of retinoic acid and of the aromatic analogue 1430 was studied at concentrations of 0·35 and 0·75 μg/ml medium. The uptake of the tracer was determined in explants pretreated with MCA for 10 days and transferred either to control medium or medium containing either of the 2 retinoids. Fig. 11 shows that 25% of the cells were labelled in explants carried on in control medium and that both retinoids halved this value. At the concentrations used, the reduction did not seem to be dose-related.

### DISCUSSION

MCA induces hyperplasia, dysplasia and squamous metaplasia of the prostatic epithelium, which persist after withdrawal of the carcinogen. Recent work (Lasnitzki, Bard and Franklin, 1975) has shown that a substantial part of MCA is retained, and presumably bound, by prostatic tissue *in vitro*, even after several changes of medium. This result may explain the persistence of the precancerous changes in the absence of added carcinogen

All the retinoids examined reduce the hyperplastic changes significantly, but the degree of inhibition varies with their structure. Thus $A_2$-retinoic acid (7057) and 13-cis-α-retinoic acid (7201) are considerably less efficient than either retinoic acid or its cyclopentenyl analogue 7699. In contrast, the aromatic compounds bearing the ethylamide (1430) or the ethylester (9359) group are highly active and even more potent inhibitors than either retinoic acid or compound 7699. Similar differences in activity have been demonstrated by Bollag (1974, 1975), who found that the 2 aromatic analogues caused regression of DMBA-induced mouse papillomas at considerably lower concentrations than retinoic acid.

The autoradiographic study shows that retinoic acid, and the aromatic analogue 1430, depress the uptake of $^3$H-thymidine by the prostatic epithelium and suggests that the reversal of hyperplasia is, at least partially, mediated *via* a reduction of DNA synthesis.

In addition to counteracting epithelial cell proliferation, most of the compounds abolish or modify the squamous changes normally associated with the hyperplasia. Retinoic acid, its cyclopentenyl analogue 7699 and 2 of the aromatic analogues, fully restore the secretory character of the epithelium; the hyperplastic epithelium, if present, is composed of columnar cells or forms adenoma-like structures. $A_2$-retinoic acid also suppresses the squamous transformation, but does not induce secretory epithelium: instead the cells remain undifferentiated and present a basal-cell-like appearance. The aromatic analogue 1670, bearing a terminal carboxyl group, is the exception and does not reverse the metaplasia; after a dose which produces a similar degree of growth inhibition to retinoic acid, the hyperplastic epithelium remains squamous. These results suggest that the inhibition of cell growth may not be directly linked with the capacity of the retinoids to restore normal epithelial differentiation.

Vitamin A is necessary for the support of growth and life (Thompson, Howell and Pitt, 1964). Of the compounds studied here, retinoic acid possesses biological growth-promoting activity, while the cyclopentenyl analogue 7699, and the aromatic analogue 1430, have virtually none (Goodman *et al.*, 1974; Bollag, personal communication). Since all 3 compounds inhibit the MCA-induced hyperplasia, it can be concluded that their anti-carcinogenic properties are independent of their biological growth-promoting activity.

A substantial part of the carcinogen remains bound to the tissue after transfer to control medium (Lasnitzki *et al.*, 1975) and the retinoids may break this bond. If so, a greater amount of free carcinogen would be released from tissue exposed to the vitamins. This expectation was not realized: the release of tritiated MCA from mouse prostatic tissue *in vitro* was of the same order in the absence or presence of retinoic acid (Lasnitzki and Goodman, 1974).

*In vitro* studies (Dingle and Fell, 1963) have shown that vitamin A promotes the release of lysosomal enzymes. This action is unrelated to the anti-carcinogenic properties of the vitamin (Lasnitzki and Goodman, 1974) but is most likely to account for its toxicity. In cartilage, the process results in the degradation of the ground substance and loss of metachromasia. In a companion study (Bard and Lasnitzki, in preparation) the toxicity of various retinoids has been investigated using the release of $S^{35}$-sulphate and loss of metachromasia in rabbit ear cartilage *in vitro* as a measure of toxicity. The toxicity was found not to be related to the ring structure of the compounds but to their terminal group, and retinoids with a carboxyl group, such as retinoic acid and the aromatic analogue 1670, provoked a high sulphate release, while analogues with an ethylester or ethylamide group were much less active.

There is recent evidence that in various mammalian and avian tissues, retinoic acid, the cyclopentenyl analogue 7699 and the aromatic analogues are bound to specific proteins (Chityl and Ong, 1976; Sani and Hill, 1976) and that the degree of binding is related to the anticarcinogenic property of the compounds and their ability to inhibit keratinization. The stability of the vitamin–protein complex suggests that it may be a tissue receptor (Sani and Hill, 1976). It is tempting to speculate that like the steroid hormone receptor complex the vitamin–protein complex may also be transported into the nucleus and bound to nuclear chromatin. In this context it is interesting that in the prostrate gland, vitamin A and testosterone show certain similarities of action. Firstly, like the hormone, the retinoids applied without the carcinogen maintain epithelial height and secretory activity of the prostatic epithelium (Lasnitzki and Goodman, 1974). Secondly and more importantly, testosterone has been found to inhibit or suppress MCA-induced hyperplasia in the rat prostate *in vitro* (Lasnitzki 1965, 1970). Thus vitamin A resembles a hormone in its ability to maintain secretory epithelia and inhibit hyperplasia, and the hormonal aspects of vitamin A activity await further study.

I am greatly indebted to Dr N. T. Pollitt and Dr W. Bollag of Hoffmann–La-Roche, Welwyn Garden City, England and Basle, Switzerland, for the gift of the vitamin A compounds used in these experiments. I should like to thank Liesbet Brown for skilled technical assistance, Peter Lancaster for the preparation of the histograms and David Bard for help with the microphotography.

The work was supported by the Cancer Research Campaign.

### REFERENCES

Bollag, W. (1972) Prophylaxis of Chemically Induced Benign and Malignant Epithelial Tumours by vitamin A Acid (Retinoic Acid). *Eur. J. Cancer*, 8, 689.

BOLLAG, W. (1974) Therapeutic Effect of an Aromatic Retinoic Acid Analog on Chemically Induced Skin Papillomas and Carcinomas in Mice. *Eur. J. Cancer*, **10**, 731.

BOLLAG, W. (1975) Therapy of Epithelial Tumours with an Aromatic Retinoic Acid Analog. *Chemotherapy*, **21**, 236.

BOLLAG, W. & OTT, F. (1970) Retinoic Acid. Topical Treatment of Senile or Actinic Ketatosis and Basal Cell Carcinomas. *Agents and Actions*, **1**, 172.

CHU, E. W. & MALMGREN, R. A. (1965) An Inhibitory Effect of Vitamin A on the Induction of Tumours of Forestomach and Cervix in the Syrian Hamster by Carcinogenic Polycyclic Hydrocarbons. *Cancer Res.*, **25**, 884.

CHYTIL, F. & ONG, D. E. (1976) Mediation of Retinoic Acid-induced Growth and Anti-tumour Activity. *Nature, Lond.*, **260**, 49.

CONE, M. V. & NETTESHEIM, P. (1973) Effects of Vitamin A on 3-Methylcholanthrene Induced Squamous Metaplasia and Early Tumours of the Respiratory Tract of Rats. *J. natn. Cancer Inst.*, **50**, 1599.

DELUCA, L. & WOLF, G. (1968) Effect of Vitamin A on Mucopolysaccharides of Lung Tissue. *Archs Biochem. Biophys.*, **123**, 1.

DINGLE, J. T. & FELL, H. B. (1963) Studies on the Mode of Action of Excess Vitamin A. 6 Lysosomal Protease and the Degradation of Cartilage Matrix. *Biochem. J.*, **87**, 403.

EVARD, J. P. & BOLLAG, W. (1972) Konservative Behandling der rezidivierenden Harnblasenpapillomatose mit Vitamin A Saüre. *Schweiz. med. Wschr.*, **102**, 1880.

GOODMAN, D. S., SMITH, J. E., HEMBRY, R. M. & DINGLE, J. T. (1974) Comparison of the Effects of Vitamin A and its Analogs upon Rabbit Ear Cartilage in Organ Culture and upon Growth of the Vitamin A Deficient Rat. *J. Lipid Res.*, **15**, 406.

HAYES, K. C., MCCOMBS, H. L. & FAHERTY, T. P. (1970) The Fine Structure of Vitamin A Deficiency. I. Parotid Duct Metaplasia. *Lab. Invest.*, **22**, 81.

KAUFMAN, D. G., BAKER, M. S., SMITH, J. M., HENDERSON, W. R., HARRIS, C. C., SPORN, M. B. & SAFFIOTTI, U. (1972) RNA Metabolism in Tracheal Epithelium: Alteration in Hamsters Deficient in Vitamin A. *Science, N.Y.*, **177**, 1105.

LASNITZKI, I. (1955) The Influence of A-hypervitaminosis on the Effect of 20-methylcholanthrene on Mouse Prostate Glands *in Vitro. Br. J. Cancer*, **9**, 434.

LASNITZKI, I. (1965) Action and Interaction of Hormones and Methylcholanthrene on the Ventral Prostate Gland of the Rat *in Vitro. J. natn. Cancer Inst.*, **35**, 339.

LASNITZKI, I. (1969) The Effect of Actinomycin D and Methylcholanthrene on the Cytology and RNA and Protein Synthesis in Prostatic Epithelium Grown *in Vitro. Cancer Res.*, **29**, 318.

LASNITZKI, I. (1970) The Action of Testosterone and its Metabolites on the Rat Prostate Gland in Organ Culture. In: *Advances in the study of the prostate.* London: Heineman Medical Books Ltd. p. 65.

LASNITZKI, I., BARD, D. R. & FRANKLIN, H. R. (1975) 3-Methylcholanthrene Uptake and Metabolism in Organ Culture. *Br. J. Cancer*, **32**, 219.

LASNITZKI, I. & GOODMAN, D. S. (1974) Inhibition of the Effects of Methylcholanthrene on Mouse Prostate in Organ Culture by Vitamin A and its Analogs. *Cancer Res.*, **34**, 1564.

MORGAN, J. F., MORTON, H. J. & PARKER, R. C. (1950) Nutrition of Animal Cells in Tissue Culture. *Proc. Soc. exp. Biol. Med.*, **73**, 1.

PRUTKIN, L. (1973) Antitumour Activity of Vitamin A acid and Fluorouracil Used in Combination on the Skin Tumour, Keratoacanthoma. *Cancer Res.*, **33**, 128.

SAFFIOTTI, U., MONTESANO, R., SELLAKUMAR, A. R. & BORG, S. A. (1967) Experimental Cancer of the Lung. Inhibition by Vitamin A of the Induction of Tracheo Bronchial Squamous Metaplasia and Squamous Cell Tumours. *Cancer, N.Y.*, **20**, 857.

SANI, S. B. & HILL, O. L. (1976) A Retinoic Acid Binding Protein from Chick Embryonic Skin. *Cancer Res.*, **36**, 409.

THOMPSON, J. N., HOWELL, J. M. & PITT, G. A. J. (1964) Vitamin A and Reproduction in Rats. *Proc. R. Soc. London, Ser. B*, **159**, 510.

WOLBACH, S. B. & HOWE, P. R. (1925) Tissue Changes Following Deprivation of Fat Soluble A Vitamin. *J. exp. Med.*, **42**, 753.